# Factors affecting discrepancies between scorers in manual sleep spindle detections in single-channel electroencephalography in young adult males

**DOI:** 10.3389/frsle.2024.1427540

**Published:** 2024-11-18

**Authors:** Yukari Tamamoto, Tatsuro Fujie, Kouichi Umimoto, Hideo Nakamura

**Affiliations:** ^1^Division of Biomedical Engineering, Graduate School of Biomedical Engineering, Osaka Electro-Communication University, Osaka, Japan; ^2^Clinical Engineering Department, Osaka Gyoumeikan Hospital, Osaka, Japan; ^3^Department of Clinical Engineering, Faculty of Medical Science Technology, Morinomiya University of Medical Sciences, Osaka, Japan; ^4^Department of Medical Sciences, Faculty of Biomedical Engineering, Osaka Electro-Communication University, Osaka, Japan; ^5^Department of Health Promotion and Sports Science, Faculty of Biomedical Engineering, Osaka Electro- Communication University, Osaka, Japan

**Keywords:** sleep EEG, sleep spindle, manual detection, discrepancies between individuals, automatic detection

## Abstract

Here, we aimed to clarify the factors that cause individual differences in manual spindle detection during sleep by comparing it with automatic detection and to show the limitations of manual detection. Polysomnography (PSG) signals were recorded from ten young male participants, and sleep stages were classified based on these signals. Using time-frequency analysis, we detected sleep spindles from the single-channel electroencephalography (EEG) of C4-A1 within the same PSG data. Our results show a detailed accuracy evaluation by comparing the two skilled scorers' outputs of automatic and manual sleep spindle detection and differences between the number of sleep spindle detections and spindle time length. Additionally, based on automatic detection, the distribution of Cohen's kappa for each scorer quantitatively showed that individual scorers had detection thresholds based on EEG amplitude. Conventionally, automatic detection has been validated using manual detection outputs as the criterion. However, using automatic detection as the standard and analyzing the manual detection outputs, we quantitatively showcased the differences in individual scorers. Therefore, our method offers a quantitative approach to examining factors contributing to discrepancies in sleep spindle detection. However, individual differences cannot be avoided when using manual detection, and automatic detection is preferable when analyzing data to a certain standard.

## 1 Introduction

Manual sleep analysis remains the prevailing standard despite substantial advancements and research in automated sleep analysis. Sleep analysis is usually performed in 30-s epochs corresponding to sleep stages called polysomnography (PSG). Lee et al. (2022) surveyed 101 references and showed the robustness of the reliability of manual determination of sleep stages using PSG. However, they also showed low accuracy for specific sleep stages, such as sleep stage N1. Notably, numerous publications have explored sleep analysis methods using a few signals and evaluating their accuracy by comparing them with PSG. PSG requires ~1,000 sleep stage classifications for a single night of data. Even for epoch units, Lee et al. report that the unification of criteria among scorers is difficult. Therefore, it is necessary to reduce the effort of manual judgment; however, sleep research should also consider using comparable indices that do not depend on the datasets. Thus, sleep analysis techniques using objective indices based on physiological mechanisms should be established.

During sleep, electroencephalography (EEG) exhibits several characteristic waves. Typical examples include the delta, alpha, and K-complex waves, which occur during slow wave sleep, eye closure, and sleep stage N2, respectively. Sleep spindles manifest during sleep stage N2 and are characterized by small amplitudes and durations of >0.5 s. Animal experiments have revealed that sleep spindles during light sleep are attributed to periodic inhibition of thalamocortical circuits by neural activity from the thalamic reticular nucleus, resulting in rhythmic activity (Llinás and Steriade, 2006). The American Academy of Sleep Medicine (AASM) manual states that “train of distinct waves with frequency 11–16 Hz (most commonly 12–14 Hz) with a duration > 0.5 s, usually maximal in amplitude using central derivations” (Berry et al., 2016). Furthermore, it has been reported that during light sleep, external stimuli such as sensory, visual, and auditory stimuli can induce sleep spindles and increase their density and length (Sato et al., 2007). These reports suggest a correlation with cranial nerve function activity levels. Therefore, given the physiological significance of sleep spindle detection and its properties outlined in the reports above, sleep spindle detection technology is promising for advancing our understanding of sleep. Furthermore, sleep spindle density is reportedly associated with various physiological and pathological conditions and diseases, indicating its potential as an indicator. In patients with Parkinson's disease, sleep spindle density is reduced, sleep spindle length is prolonged, sleep spindle frequency is reduced, and maximum peak-to-peak amplitude is increased compared with controls. Additionally, an increase in maximum peak-to-peak amplitude has been reported compared with controls (Christensen et al., 2015). Furthermore, sleep spindle density has been associated with the progression of the pathology of narcolepsy, suggesting its association with cranial nerve function activity levels (Christensen et al., 2017). Additionally, sleep spindle density is reportedly associated with the duration of sleep stage N2 and recovery. Furthermore, it is associated with intelligence, suggesting its potential as an intelligence marker and a marker for assessing cognitive dysfunction and dementia (Sato et al., 2007). The sleep spindle density increased by an average of 30% during the first 90 min of sleep onset after learning in 11 of 13 participants (Gais et al., 2002). In addition, it has been associated with daytime activity and implicated in memory consolidation (Laventure et al., 2016). Therefore, if sleep spindle density and length can be accurately measured, the state of cognitive function and memory consolidation can then be examined.

Detecting individual sleep spindle periodic waveforms of >0.5 s as with PSG proves to be time-consuming and expensive, thus hindering sleep research involving large-scale data analysis. Therefore, it is essential to promote automatic sleep spindle detection methods. Notably, some reports have shown the accuracy of automatic detection of sleep spindles. Wendt et al. (2015) concluded that automatic motion detection of sleep spindles has sufficient test-retest reliability. However, Wendt et al. also argue that there is a challenge to the acceptance of automatic detection of sleep spindles as a standard. Therefore, the sources of discrepancies between automatic and manual detection must be identified and resolved. This issue is a necessary consideration when establishing automatic sleep spindle detection technology. In addition, O'Reilly and Nielsen (2015) conducted a detailed investigation into the accuracy of sleep spindle detection by scorers across multiple databases, providing valuable insights for sleep spindle detection. They also provided essential implications for sleep spindle detection. First, they showed that there are significant confounding factors between scorers and databases. They also stated that sleep spindles are relatively sparse phenomena within the EEG signal and highlighted that sensitivity and specificity alone are insufficient for evaluating accuracy. Therefore, they recommended the use of comprehensive statistics such as the F1-score and Cohen's kappa.

Furthermore, the inherent discrepancies among scorers in manual detection add complexity to sleep research (Kaulen et al., 2022). Thus, solving the above problems and developing a standardized automatic detection algorithm could reduce the cost of sleep spindle detection, increase time efficiency, and ensure reproducibility. Therefore, there is a need to determine the causes of discrepancies in manual detection and feed them back into the automatic detection technology.

Consequently, we aimed to clarify the factors that cause individual differences in manual spindle detection during sleep by comparing it with the automatic detection method using the Complex Demodulation Method (CDM) and show the limitations of manual detection.

## 2 Methods

### 2.1 Participants

Ten healthy young male participants were enrolled in this study, and their characteristics are presented in Table 1. The participants had a mean age of 21.3 ± 0.7 years [mean ± standard deviation (SD)] and a mean body mass index of 24.3 ± 3.8 kg/m^2^. The participants had no history associated with sleep disorders. Furthermore, their total sleep time (TST) and sleep efficiency were 6:53:03 ± 0:51:42 and 84.9 ± 10.5%, respectively. The Apnea Hypopnea Index (AHI) was 2.3 ± 1.5. The participants were instructed to avoid intense exercise and abstain from alcohol, drugs, and caffeine intake starting from the day before the experiment. They were provided with detailed information about the experiment, including its procedures, potential benefits, and risks.

**Table 1:** The characteristics and sleep parameters of the 10 participants.

**ID**	**Age (years)**	**Height (cm)**	**Weight (kg)**	**BMI (kg/m^2^)**	**AHI**	**TST (hh:mm:ss)**	**Sleep efficiency (%)**
1	21	165	79.0	29.0	4.1	6:13:30	76.7
2	21	177	63.0	20.1	0.6	6:31:30	80.5
3	21	163	70.9	26.7	3.0	7:45:00	95.7
4	21	168	55.1	19.5	1.0	7:16:00	89.4
5	20	169	62.7	22.0	3.9	7:39:00	95.2
6	21	166	63.4	23.0	0.4	6:50:00	85.0
7	22	173	77.7	26.0	1.2	7:33:59	93.4
8	22	172	86.6	29.3	2.9	7:41:30	92.6
9	22	169	78.5	27.8	4.4	6:11:30	77.1
10	22	175	61.5	20.1	1.4	5:08:30	63.4
	21.3 ± 0.7	169.7 ± 4.5	69.8 ± 10.2	24.3 ± 3.8	2.3 ± 1.5	6:53:03 ± 0:51:42	84.9 ± 10.5
							(Mean ± SD)

### 2.2 PSG recording and scoring

A SOMNOscreenTM^®^ from SOMNOmedics was used as the PSG recording device. The weight of the device itself was 206 g. The sensors were attached to the participants, and they included EEG, electrooculogram, electromyography (EMG) of the mentalis muscle, airflow, snore, ECG, thoracic and abdominal movement, SpO2, and EMG of the anterior tibialis muscle. The sampling frequency for each signal can be adjusted from 4 to 512 Hz. The sampling frequency of the EEG was set at 256 Hz.

The participants were instructed to arrive at the laboratory at 21:00. They filled out a questionnaire and provided biometric information, such as height and weight. Subsequently, the various sensors for PSG were attached. Once the preparation was completed, the participants commenced their sleep period in the designated recording room. The bedtime ranged from 22:00 to 23:00, and the waking time ranged from 6:00 to 7:00. The total sleep duration exceeded 8 h for all participants. Notably, the participants were permitted to move freely during recording owing to the separation between the transmitter and the receiver. Their biomedical signals were recorded on a CompactFlash card inserted into the transmitter and transmitted through Wi-Fi to the monitoring room. When it was time to wake up, the examiner asked the participants to get up, and the recording was stopped.

An experienced scorer with over 10 years of analysis expertise classified the sleep stage from the PSG signals. The analysis software used was DOMINO^®^ from SOMNOmedics. The rules for scoring the sleep stages adhered to the guidelines outlined in the AASM Manual version 2.3 for the Scoring of Sleep and Associated Events (Berry et al., 2016).

### 2.3 The procedures of automatic and manual decision for sleep spindle

First, two skilled scorers (Scorer A: 10 years of experience in sleep EEG analysis in a medical institution and Scorer B: 7 years of experience in sleep EEG analysis in a medical institution) performed manual detection of sleep spindles. The manual detection process followed the guidelines outlined in the AASM scoring manual (Berry et al., 2016). The DOMINO^®^ software displayed all signals recorded within a 30-s epoch on the computer screen to detect the sleep spindles. Sleep spindle onset and endpoints were determined through mouse operation, and manual detections used the single EEG channel at C4-A1.

Automatic detection extracted sleep spindles from the same single-channel EEG signals as manual detections using CDM, a time-frequency analysis method for evaluating the amplitude of a specific frequency in a signal (Bloomfield, 1976; Zeitlhofer et al., 1997; De Gennaro and Ferrara, 2003). The CDM was used for the automatic extraction of sleep spindles because, unlike the short-time Fourier transform, the CDM has the advantage of being able to freely select time and frequency resolution within the time-frequency analysis. Notably, there are several reports on using CDM to detect sleep spindles (Kumar et al., 1979; Hao et al., 1992), and it has recently been used to automatically detect sleep spindles (Ray et al., 2015).

As discussed in Section 1, the AASM manual states, “train of distinct waves with frequency 11–16 Hz (most commonly 12–14 Hz) with a duration > 0.5 s, usually maximal in amplitude using central derivations” (Berry et al., 2016). Therefore, the frequency bandwidth of sleep spindles is generally recognized as 11–16 Hz. However, as noted in the AASM manual, the central band of sleep spindles is 12–14 Hz. Therefore, there has been more detailed research on sleep spindles, with slow spindles around 12 Hz and fast spindles around 14 Hz, based on the differences in physiological significance (Tamaki et al., 2008). In addition, the bandwidth of 11 Hz overlaps with the alpha waves, and 16 Hz overlaps with the beta waves; therefore, limiting the frequency to 12–14 Hz reduces the influences from alpha and beta waves. Thus, in the automatic detection in this study, we decided to extract sleep spindles in the 12–14 Hz range.

**Figure 1:**
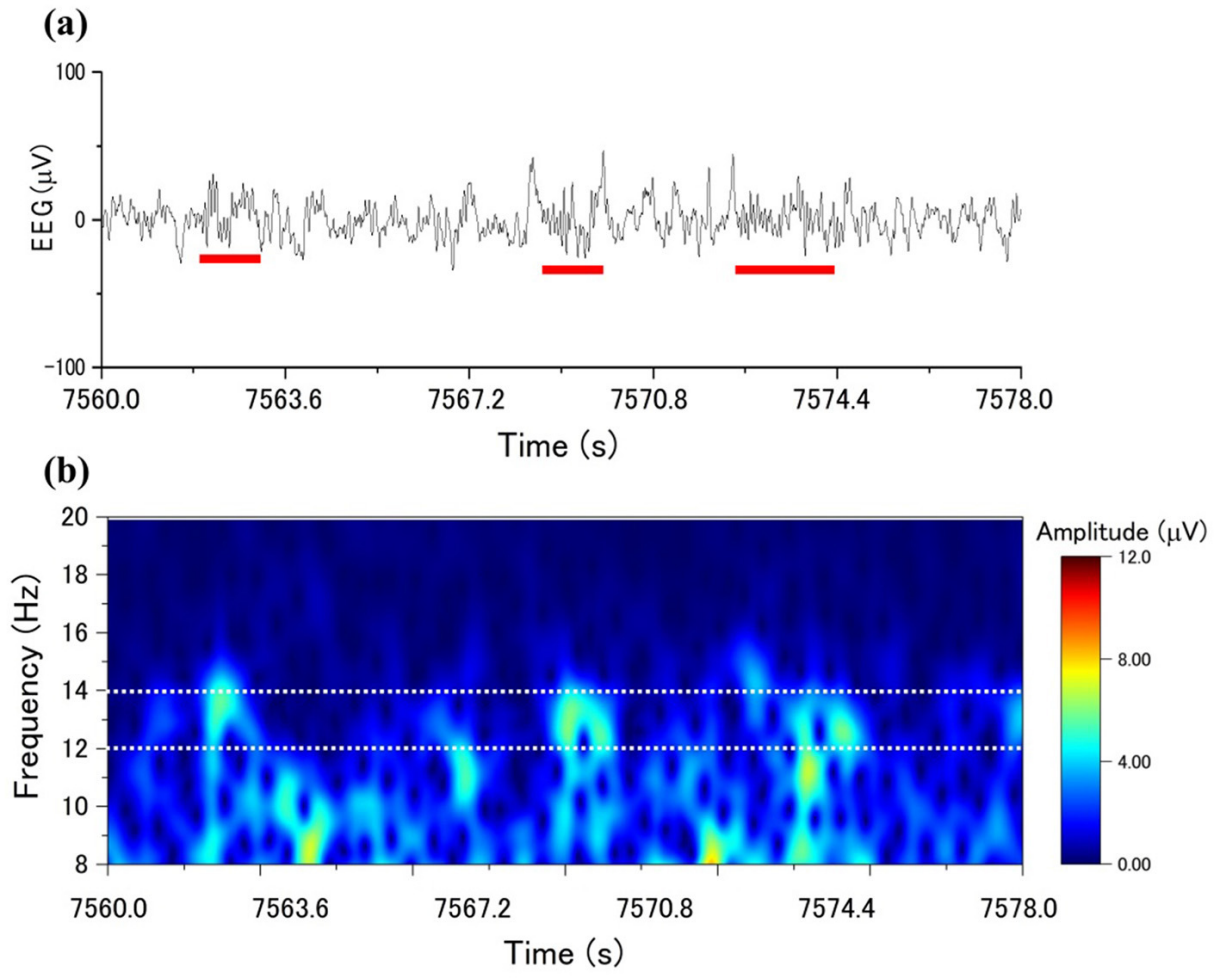
CDM amplitude distributions to sleep spindles: **(a)** an EEG with spindle occurrence marked by a red line, **(b)** the time-frequency distribution with CDM from the EEG in **(a)**.

The EEG signals from the electrodes of C4-A1, the parietal EEG, were employed to analyze the EEG using CDM. Figure 1a shows the sleep spindles in a segment of a single-channel EEG, whereas Figure 1b exhibits the spectrogram obtained by applying CDM to the occurrences of the sleep spindles. CDM reveals the analysis detections within the frequency range of 12–14 Hz (the range between the white dotted lines in Figure 1b), corresponding to the appearance of sleep spindles. CDM shows a bright-colored response in the region where the sleep spindles appear.

**Figure 2:**
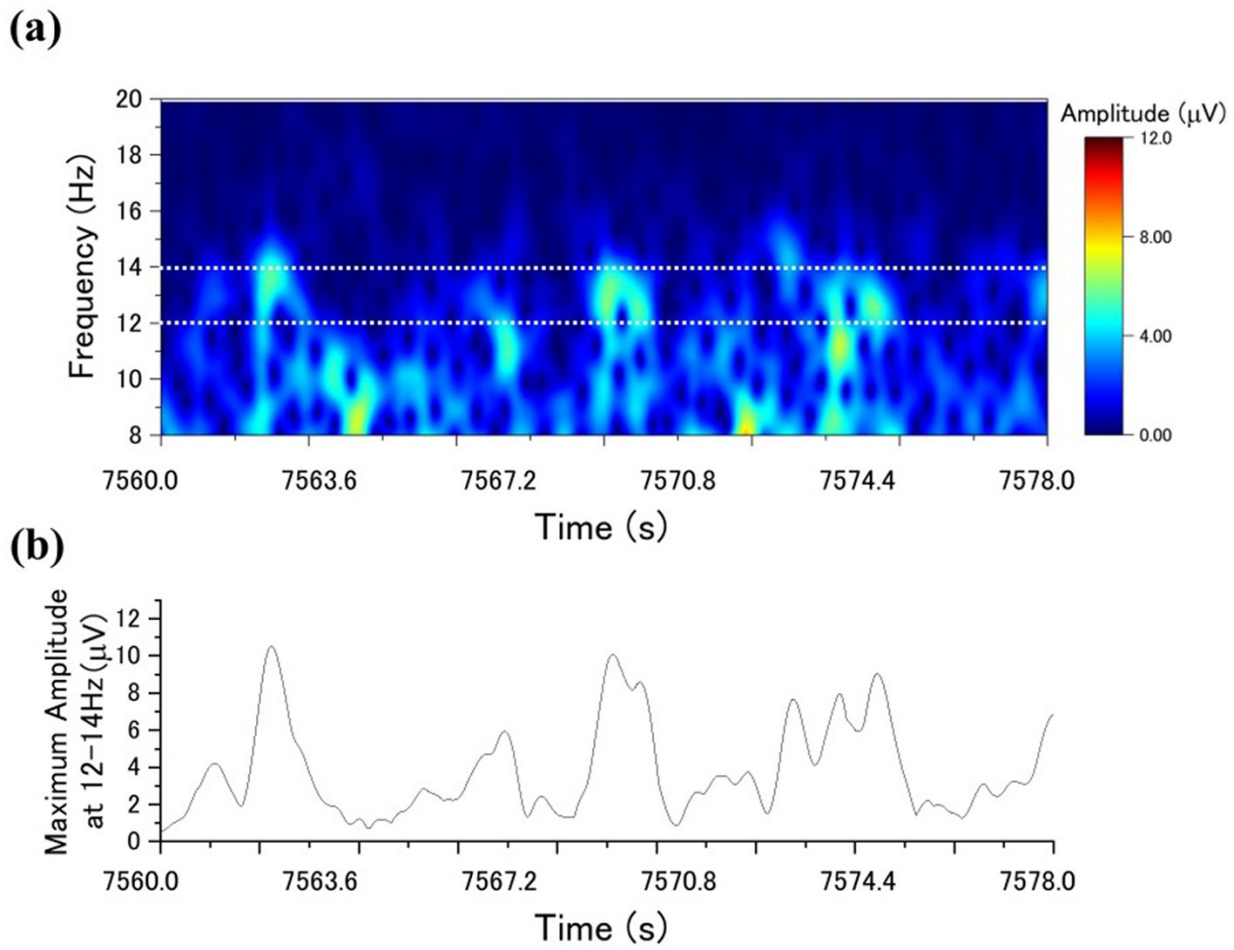
Sleep spindle detection using the CDM method: **(a)** the CDM distribution for a specific period; **(b)** the time series of the highest CDM amplitudes within the 12–14 Hz range.

The maximum amplitude value within the frequency band of 12–14 Hz was selected for each sample. Therefore, using the maximum amplitude value instead of the mean amplitude helps increase the variability of the values and facilitates the differentiation between the presence and absence of sleep spindles. Subsequently, if the maximum amplitude value, like Figure 2 of a sample, exceeds a specific threshold, the sample is classified as part of a sleep spindle. This procedure is referred to as automatic sleep spindle detection. The detailed threshold value setting is described in the Section 3.

Regarding the detected outputs, we compared manual detections from the two scorers and automatic detection, varying the threshold for detecting sleep spindles based on the CDM analysis detections. We aimed to determine the threshold value at which Cohen's kappa was maximized for each scorer. We also evaluated the Recall, Precision, and F1-score for manual detection vs. automatic detection.

Furthermore, we initially compared the manual detection outcomes between the two scorers. Cohen's kappa coefficient was calculated to assess the agreement of sleep spindle detection between the scorers. Additionally, the number of sleep spindles detected by each scorer and the duration of the detected sleep spindles were compared. A cross-tabulation evaluation was conducted, where areas identified as sleep spindles were assigned a value of 1, and those not identified were assigned a value of 0.

## 3 Results

### 3.1 Comparison between automatic and manual detection

**Figure 3:**
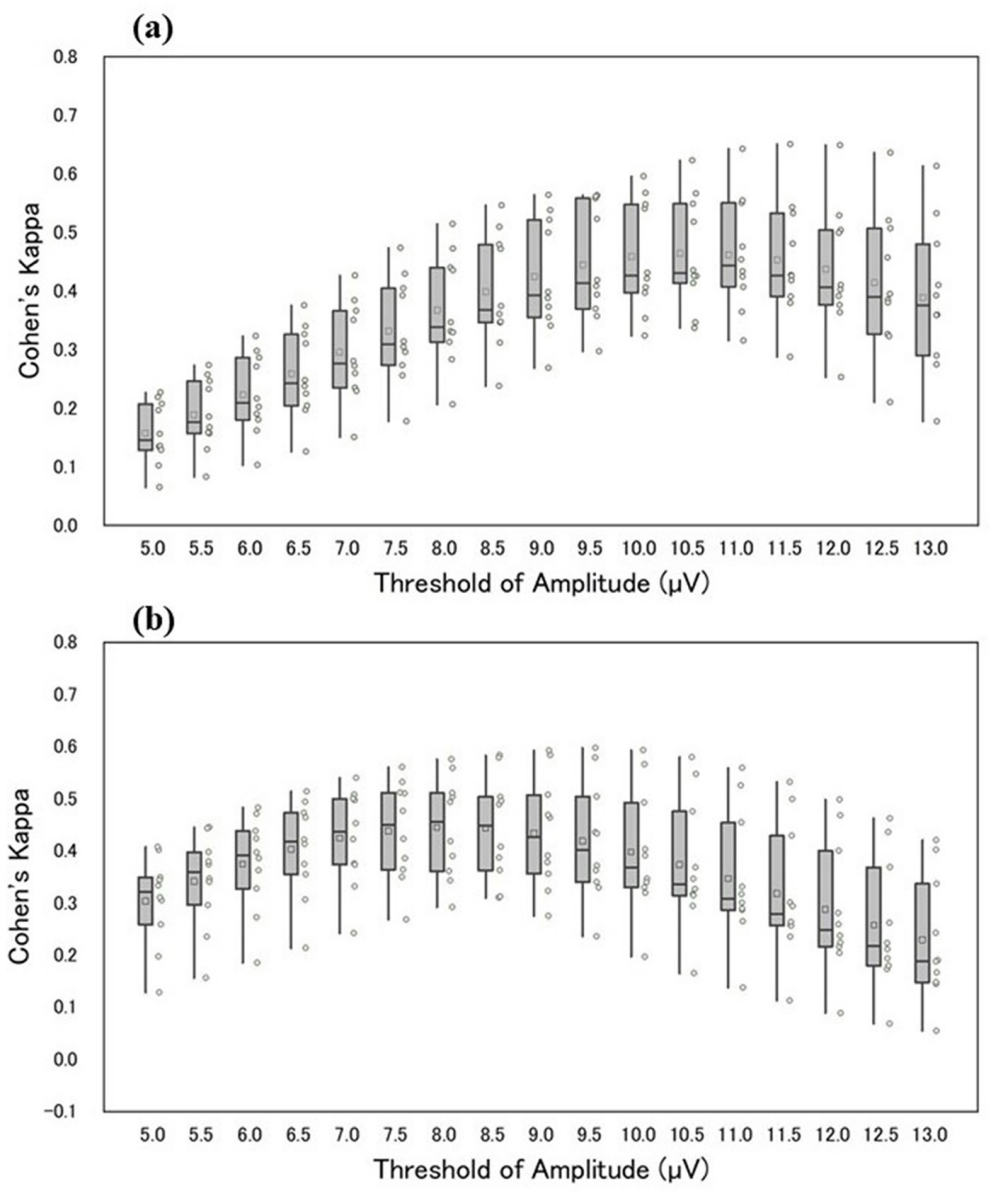
The horizontal axis of the figure shows the respective threshold values, and the vertical axis shows Cohen's kappa of manual and automatic detection **(a, b)** shows Cohen's kappa between Scorers A and B. The box-and-whisker diagram in the figure shows, from top to bottom, the maximum, third quartile, median, first quartile, and minimum values. The squares within the boxes indicate the average values, and the circles represent Cohen's kappa values for each participant.

The authors compared the outputs of manual and automatic detection of sleep spindles. Threshold values for estimating sleep spindles were determined from the maximum amplitude values in the 12–14 Hz frequency band extracted from the CDM. Initially, threshold values were set in increments of 0.5 μV within 5.0–13.0 μV. Sleep spindles for the 10 participants were detected at each threshold value. For sleep spindles detected using CDM (based on exceeding the threshold of CDM) at each threshold, the agreement between the CDM and the manually detected sleep spindles was evaluated using Cohen's kappa. Figure 3a illustrates Cohen's kappa values for Scorer A in manual and automatic detection, whereas Figure 3b displays Cohen's kappa values for Scorer B. The median Cohen's kappa value for manual detection at each automatic detection threshold was greater than that for Scorer A when the threshold was set at 11.0 μV and for Scorer B when the threshold was set at 8.0 μV. Compared with Scorer A, Scorer B tended to detect sleep spindles of smaller amplitude. Table 2 shows the details of the number and duration of each detection, with the threshold set at 11.0 and 8.0 μV, respectively.

**Table 2:** The sleep spindle decision by scorers A and B for the participants.

**ID**	**Count**		**Average duration (s)**		**Maximum duration (s)**		**Minimum duration (s)**	
	**Automatic detection threshold 11.0** μ**V**	**Automatic detection threshold 8.0** μ**V**	**Automatic detection threshold 11.0** μ**V**	**Automatic detection threshold 8.0** μ**V**	**Automatic detection threshold 11.0** μ**V**	**Automatic detection threshold 8.0** μ**V**	**Automatic detection threshold 11.0** μ**V**	**Automatic detection threshold 8.0** μ**V**
1	242	1,019	0.72	0.81	1.95	2.60	0.50	0.50
2	519	1,618	0.79	0.88	3.13	4.03	0.50	0.50
3	909	1,945	0.87	0.97	2.22	3.75	0.50	0.50
4	886	2,257	0.72	0.82	1.80	4.26	0.50	0.50
5	328	992	0.73	0.82	1.71	2.21	0.50	0.50
6	260	1,112	0.69	0.78	1.64	3.44	0.50	0.50
7	478	1,334	0.79	0.85	3.89	7.02	0.50	0.50
8	989	2,066	0.86	0.99	2.29	4.38	0.50	0.50
9	123	618	0.69	0.76	1.68	2.75	0.50	0.50
10	432	1,190	0.86	0.91	4.26	7.00	0.50	0.50
Mean ± SD	516.6 ± 308.0	1415.1 ± 534.9	0.77 ± 0.07	0.86 ± 0.08	2.46 ± 0.96	4.14 ± 1.67	0.50 ± 0.00	0.50 ± 0.00
*p*-value	0.87	0.49	0.002^**^	0.00001^‡^	0.06	0.0026^‡^	0.0096^**^	2.6E-12^‡^

^**^p < 0.01 vs. scorer A, ^‡^*p* < 0.01 vs. scorer B in this table with the paired *t*-test.

Two extraction thresholds were adopted: 11.0 μV, which had the highest Cohen's kappa with the extraction by Scorer A, and 8.0 μV, which had the highest Cohen's kappa with the extraction by Scorer B.

The authors also evaluated the agreement between scorers A and B and the automatic detection; a threshold for the CDM analysis was set at 10 μV based on the general definition. Consequently, Cohen's kappa between Scorer A and automatic analysis was 0.48 ± 0.10, and that between Scorer B and automatic analysis was 0.34 ± 0.14. Furthermore, as shown in Table 3, the F1-score between Scorer A and automatic detection was 0.49 ± 0.10, and that between Scorer B and automatic detection was 0.36 ± 0.14.

**Table 3:** Precision, recall, F1-score, and Cohen's kappa for manual detection by two skilled scorers' decisions and for automatic decisions in ten participants.

	**Precision**	**Recall**	**F1-score**	**Cohen's kappa**
Scorer A vs. auto	0.51 ± 0.15	0.54 ± 0.18	0.49 ± 0.10	0.53 ± 0.09
Scorer B vs. auto	0.64 ± 0.14	0.27 ± 0.14	0.36 ± 0.14	0.48 ± 0.09
Scorer A vs. scorer B	0.29 ± 0.09	0.79 ± 0.10	0.42 ± 0.10	0.41 ± 0.10
				(Mean ± SD)

### 3.2 Inter-scorers comparison

**Figure 4:**
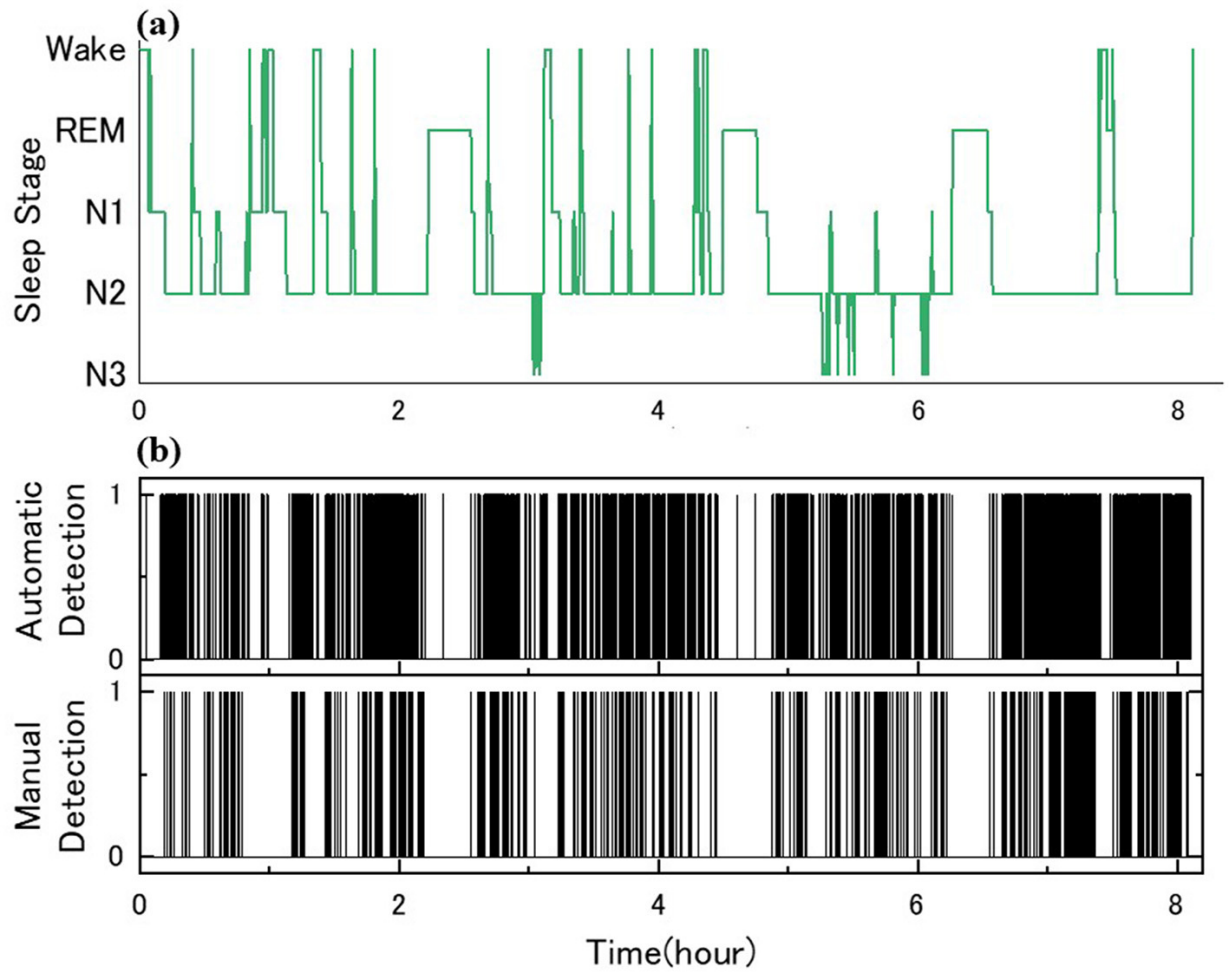
Sleep spindle detection and comparison for ID 7: **(a)** sleep architecture diagram with PSG; **(b)** the binary decisions on the automatic and manual spindle detection, 1 for presence and 0 for absence.

Two skilled scorers performed manual detection of sleep spindles for the 10 participants. Examples of sleep stage determination by Scorer A and manual and automatic detection are shown in Figure 4. The Cohen's kappa for the two detections yielded moderately consistent results at 0.41 ± 0.10. Table 4 shows the details of the number of detection counts and times for each detection. The average number of sleep spindles detected was 537.9 ± 267.0 and 1,275.9 ± 347.5 for Scorers A and B, respectively, in the 10 participants, with Scorer B detecting ~2.4 times more sleep spindles than Scorer A. The average duration of the detected sleep spindles was 0.96 ± 0.15 s for Scorer A and 1.04 ± 0.05 s for Scorer B, with no significant difference in the paired *t*-test (*p* = 0.15). The maximum duration of the detected sleep spindles was 1.79 ± 0.40 s for Scorer A and 2.27 ± 0.27 s for Scorer B in the average of 10 male participants, and the paired *t*-test showed a significant tendency for Scorer B to detect even longer sleep spindles (*p* < 0.05). The minimum duration of the detected sleep spindles was 0.54 ± 0.04 s for Scorer A and 0.51 ± 0.00 s for Scorer B. Furthermore, the minimum duration for Scorer B was also the same value; thus, the SD was 0. Therefore, the *t*-test could not be applied. This may be because the AASM rules define sleep spindles as ≥0.5 s, and Scorers intentionally extracted spindles > 0.5 s.

**Table 4:** The sleep spindle decision by scorers A and B for the participants.

**ID**	**Count**		**Average duration (s)**		**Maximum duration (s)**		**Minimum duration (s)**	
	**Scorer A**	**Scorer B**	**Scorer A**	**Scorer B**	**Scorer A**	**Scorer B**	**Scorer A**	**Scorer B**
1	318	974	1.18	1.03	1.89	2.21	0.63	0.51
2	281	848	1.18	0.99	2.64	1.98	0.59	0.51
3	377	1,657	1.13	1.03	2.24	2.87	0.55	0.51
4	1,113	1,959	0.93	0.95	1.57	2.04	0.55	0.51
5	500	1„292	0.92	1.04	1.73	2.11	0.51	0.51
6	733	1,274	0.85	1.02	1.65	2.28	0.51	0.51
7	492	1,407	0.83	1.05	1.38	2.08	0.51	0.51
8	799	1,359	0.95	1.15	1.89	2.36	0.51	0.51
9	303	899	0.82	1.10	1.34	2.25	0.51	0.51
10	463	1,090	0.83	1.02	1.54	2.52	0.51	0.51
	537.9 ± 267.0	1,275.9 ± 347.5	0.96 ± 0.15	1.04 ± 0.05	1.79 ± 0.40	2.27 ± 0.27	0.54 ± 0.04	0.51 ± 0.00
								(Mean ± SD)

Table 5 shows a cross-tabulation table of sleep spindle detection for the 10 participants. Notably, of the total recording time of 291,887 s for the 10 participants, there were 4,027.8 s during which both scorers identified the presence of a sleep spindle. Additionally, there were 1,030.9 s when Scorer A detected a sleep spindle while Scorer B did not, and 9,172.1 s when Scorer B detected a sleep spindle while Scorer A did not.

**Table 5:** The cross-tabulation of the sleep spindle decisions between scorers A and B.

		**Scorer B**					
		**1**		**0**		**Total**	
Scorer A	1	4,027.8 s	(1.4%)	1,030.9 s	(0.4%)	5.058.8 s	(1.7%)
	0	9,172.1 s	(3.1%)	277,656.2 s	(95.1%)	286,828.2 s	(98.3%)
	Total	13,199.9 s	(4.5%)	278,687.1 s	(95.5%)	291,887.0 s	(100.0%)

## 4 Discussion

The authors agree with the opinions of O'Reilly et al. and, accordingly, present the statistics such as Cohen's kappa and F1-score from our study in Table 3. O'Reilly et al. also raised concerns about the reliability of scorer-based manual scoring as the definitive gold standard. In this study, we objectively highlight the noted problems by quantifying individual differences in manual detections. The findings in Figure 3 indicate that the outcomes of sleep spindle detection by the two scorers, using the same automatic detection method, are amplitude-dependent and reflect variations in scorer individuality. Moreover, Figure 3 provides statistical insights into the number of sleep spindle detections and sleep spindle lengths between scorers. Notably, Scorer B detected more sleep spindles than Scorer A in all participants, highlighting the influence of amplitude as a criterion.

Table 3 presents the precision, recall, and F1 scores for Scorers A and B. According to the paired *t*-test (*p* < 0.05), Scorer B's F1-score was significantly lower than that of Scorer A. Figure 3 highlights an amplitude-dependent variation between the two scorers, potentially contributing to the observed difference in F1-scores. Precision, recall, and F1-score were evaluated; however, none of these metrics showed high values.

In Table 5, the time intervals in which sleep spindles occurred were sparsely small compared with those in which sleep spindles did not occur due to the sample-by-sample evaluation. In addition, manual sleep spindle detection may have been affected by individual differences in the durations of the sleep spindle detections. Based on these considerations, we can conclude that Precision, Recall, and F1-score only show overall values reflecting the results of various factors and are not suitable for identifying specific discrepancy factors. Our results reveal individual differences between the two scorers by varying the amplitude threshold between automatic and manual detection. We also evaluated Cohen's kappa for each manual detection by the two scorers, with the automatic detection as the reference. From our results, we believe that it would be better not to rely solely on conventional scorer-dependent criteria. This approach can identify discrepancy causes using highly reproducible automatic algorithms.

Furthermore, we would like to discuss the issue described by Wendt et al. The issue is identifying the factors contributing to automatic and manual detection differences. Notably, many reports on accuracy verification have compared automatic detection output with manual detection. Wendt et al. present the agreement rates for manual detection within and between scorers for 24 registered polysomnography technologists: Cohen's kappa was 0.66 ± 0.07. However, according to this paper, Cohen's kappa in the sample-by-sample evaluation among the scorers was 0.52 ± 0.07. In this study, Cohen's kappa was 0.41 ± 0.10, which is a lower agreement compared with the results of Wendt et al. A possible cause of the false negatives based on automatic detection is inferred to be the difficulty in identifying during classification waveforms that overlap with K-complex and baseline fluctuations. Waveforms with large amplitude fluctuations in the frequency band close to sleep spindles, such as arousal responses, could also have been detected erroneously. As for false positives, the average duration in Tables 2–4 shows that the average duration for manual detection is significantly longer than that for automatic detection (*p* < 0.05). This means that the spindle waveforms with manual detection are significantly longer than those with automatic detection.

Consequently, the extra length of the spindle waveforms due to manual detection is detected as an error because they are longer than those due to automatic detection. This may be the reason for the lower Cohen's kappa for manual detection vs, automatic detection. The sleep spindles in this study were detected from the entire PSG of each participant, whereas in Wendt's study, sleep spindles were detected from 400 segments. The lower agreement rate may be partly due to the difference in assessment methods.

Differences in the datasets used by different research groups are a problem for further accuracy evaluation of automatic detection techniques. O'Reilly et al. have shown that there are significant confounding factors between datasets. Recently, increasing efforts have been made to make biometric data publicly available for evaluation using a standard dataset for accuracy comparisons (O'Reilly et al., 2014; Devuyst et al., 2011). Therefore, we plan to examine accuracy evaluation using these datasets in the future.

The AASM definition of a sleep spindle is a periodic waveform ranging from 12 to 14 Hz, lasting over 0.5 s. Based on this definition, Wendt et al. state that metrics such as sleep spindle density are used primarily to verify whether they adequately reflect physiological events. In this case, an event-by-event assessment of the presence or absence of sleep spindles would suffice, even if the length, onset, and end cannot be accurately estimated. Another study compared manual sleep spindle detection accuracy in event-by-event assessment to automatic sleep spindle detection (Warby et al., 2014). Notably, if only sleep spindle generation and spindle density are required, then applying event-by-event evaluation to measure sleep spindle characteristics is acceptable. However, sample-by-sample evaluation becomes necessary if sleep spindle wavelength is also required.

Consequently, sleep spindle wavelength, onset, and end time accuracy can be verified and evaluated more objectively. In this study, sleep spindle detection with sample-by-sample evaluation revealed individual differences between the scorers regarding sleep spindle duration. Therefore, it can be concluded that sample-by-sample evaluation provides a more detailed examination of the characteristics of sleep spindles compared with event-by-event evaluation.

## 5 Conclusions

In this study, PSGs from ten young male participants were recorded, and the output of an automated sleep spindle detection algorithm using CDM was compared with the outputs of two skilled scorers for the C4-A1 single-channel EEG, which was extracted from the PSGs.

When comparing Cohen's kappa for the two scorers' manual detection while varying the amplitude threshold of the automatic detection from 5.0 to 13.0 μV, we found that the amplitude threshold at which Cohen's kappa reached its maximum was 11.0 μV for scorer A and 8.0 μV for scorer B, a difference of 3.0 μV. This result indicates that the two scorers differ in their amplitude criteria for detecting sleep spindles. Individual differences in manual extraction are generally acknowledged as inevitable; however, our results quantitatively and visually revealed amplitude-dependent differences in detection between the scorers.

Currently, when evaluating the performance of automatic detection algorithms, the output of manual detection is often used as a primary standard. However, as shown in this study, we could not eliminate individual differences between judges. As our data shows, quantitatively examining the variability in manual detection results is expected to provide useful insights into the causes of discrepancies in manual analysis. Furthermore, by using highly reproducible automatic detection as a reference, more reproducible and objective measurements that are not dependent on individuals can be achieved.

## Data Availability

The datasets presented in this article are not readily available because data cannot be shared publicly due to ethical restrictions imposed by the Osaka Electro-Communication University Ethics Committee. Requests to access the datasets should be directed to Yukari Tamamoto, dl22a001@oecu.jp.
